# Teaching Computational Reproducibility for Neuroimaging

**DOI:** 10.3389/fnins.2018.00727

**Published:** 2018-10-22

**Authors:** K. Jarrod Millman, Matthew Brett, Ross Barnowski, Jean-Baptiste Poline

**Affiliations:** ^1^Division of Biostatistics, University of California, Berkeley, Berkeley, CA, United States; ^2^Berkeley Institute for Data Science, University of California, Berkeley, Berkeley, CA, United States; ^3^College of Life and Environmental Sciences, University of Birmingham, Birmingham, United Kingdom; ^4^Applied Nuclear Physics Program, Lawrence Berkeley National Laboratory, Berkeley, CA, United States; ^5^Neurology and Neurosurgery, McGill University, Montreal, QC, Canada

**Keywords:** neuroimaging, FMRI, computational reproducibility, scientific computing, statistics, education, Python language, data science

## Abstract

We describe a project-based introduction to reproducible and collaborative neuroimaging analysis. Traditional teaching on neuroimaging usually consists of a series of lectures that emphasize the big picture rather than the foundations on which the techniques are based. The lectures are often paired with practical workshops in which students run imaging analyses using the graphical interface of specific neuroimaging software packages. Our experience suggests that this combination leaves the student with a superficial understanding of the underlying ideas, and an informal, inefficient, and inaccurate approach to analysis. To address these problems, we based our course around a substantial open-ended group project. This allowed us to teach: (a) computational tools to ensure computationally reproducible work, such as the Unix command line, structured code, version control, automated testing, and code review and (b) a clear understanding of the statistical techniques used for a basic analysis of a single run in an MR scanner. The emphasis we put on the group project showed the importance of standard computational tools for accuracy, efficiency, and collaboration. The projects were broadly successful in engaging students in working reproducibly on real scientific questions. We propose that a course on this model should be the foundation for future programs in neuroimaging. We believe it will also serve as a model for teaching efficient and reproducible research in other fields of computational science.

## 1. Introduction

Few neuroimaging analyses are computationally reproducible,^1^ even between researchers in a single lab. The process of analysis is typically *ad-hoc* and informal; the final result is often the fruit of considerable trial and error, based on scripts and data structures that the author inherited from other members of the lab. This process is (a) confusing, leading to unclear hypotheses and conclusions, (b) error prone, leading to incorrect conclusions and greater confusion, and (c) an impractical foundation on which to build reproducible analyses.

Confusion, error, and lack of reproducibility are ubiquitous problems that programmers have been fighting since before “programmer” became a job title. There are widely accepted tools and processes to reduce these problems, including the Unix command line, version control, high-level readable programming languages, structured code, code review, writing tests for new code, and continuous automatic test execution.

Many researchers accept that learning these techniques is desirable, but believe that teaching them is too difficult, or would cost too much in class time that should be spent on topics specific to neuroimaging.

In the course we describe here, we tested our hypothesis that we could effectively teach *both* the tools for efficient and reproducible work *and* the principles of neuroimaging, by building the course around a substantial collaborative project, and putting the tools into immediate practice. Our emphasis on the project can be seen as a version of project-based learning (Thomas, 2000; Condliffe, 2017).

At intake, our students had little or no prior exposure to neuroimaging, or to the computational tools and process we listed above. We set them the open-ended task of designing and executing a project, which was either a replication or an extension of a published neuroimaging analysis, built from code they had written themselves. We required the analysis to be computationally reproducible; each project had to provide a short text file that gave a simple set of commands with which the grading instructor could fetch the data, run the analysis, and generate the final report, including figures.

### 1.1. Background and rationale

Between us, we have many decades experience of teaching neuroimaging analysis. Like most other teachers of imaging, we have taught traditional courses with a mixture of lectures covering the general ideas of the analysis, combined with practical workshops using one of the standard imaging software packages.

We also have many years of experience giving practical support for imaging analysis to graduate students and post docs.

Over these years of teaching and support, we have come to realize that the traditional form of teaching does a poor job of preparing students for a productive career in imaging research. It fails in two fundamental ways:

- Computation with large complex datasets is difficult and distracting; without training in standard practice to reduce this distraction, we condemn our students to a life-time of inefficient work and slow learning.
- Standard teaching assumes either that the students already understand signal processing and linear algebra, or that they do not need to understand them. In our experience, both of these assumptions are mostly false, with the result that the students do not have the foundation on which they can build further understanding.

As a result, imaging researchers usually do not have the vocabulary, understanding, or shared tools to collaborate fluently with researchers from other fields, such as statistics, engineering, or computer science.

The essential error in traditional teaching is one of emphasis; it gives priority to the overview at the expense of the intellectual and practical foundations. The assumption is that the student will either know or pick up the mathematical and practical basis, with the big picture as a framework to guide them in choosing what to learn.

In fact we find that it is rare for students to go on to learn these foundations. They usually continue as they were when they leave the course: as inefficient practitioners with little ability to reason about their analysis.

In contrast to the traditional approach, we emphasize:

- Efficient computational practice to decrease confusion, reduce error, and facilitate collaboration.
- The fundamental mathematical and statistical bases of the analysis, such as the linear model, using computational tools to build, illustrate, and explain.

We were inspired by the famous epithet of Richard Feynman, found written on his blackboard after his death: “What I cannot create, I do not understand.”^2^ We first taught the students to build code efficiently, and then we taught them how the methods worked, by building them from code. Our aim was that our students should be able to implement their own simple versions of the major algorithms they were using.

Our course put great emphasis on a final group project that accounted for more than half of the overall course grade. We did this for two reasons:

First, we believe the motivations for computational reproducibility and the difficulties of collaboration are too abstract to be meaningfully understood in the absence of a significant, concrete, group project. If a project lasts a few weeks, it is possible to remember all the steps without carefully recording and explaining them. If you have a small dataset and a handful of tiny functions, it is reasonable to throw them all in a directory and post them online. As datasets get larger and the analysis more complex, it quickly becomes impossible to keep track of what you have done without carefully organizing and recording your work. Large datasets and complex analyses are typical in research.

Second, we intended to teach the students efficient reproducible practice with the standard tools that experts use for this purpose (Millman and Pérez, 2014). Our own practice has taught us that the power of these tools only becomes clear when you use them to do substantial work in collaboration with your peers. In contrast, we have found that teaching “easy” tools that need less initial investment has the paradoxical effect of making it harder for students to move on to the more powerful and efficient tools that they will need for their daily work.

### 1.2. Hypothesis and test

Our hypothesis was that it is possible to teach the combination of standard reproducible practice and the fundamentals of neuroimaging analysis in a single undergraduate class. We considered our class to be a feasibility study for a large change in the curriculum.

Because we were doing a feasibility study, our overriding question was “can this be done?,” and we chose our teaching methods to give us the best chance of success. Our course differed in various ways from standard neuroimaging courses, including (a) teaching tools first and content later, (b) greater emphasis on fundamentals of statistical estimation and inference, (c) pervasive use of online collaboration tools for review and feedback, and (d) heavy emphasis on a substantial open-ended project as a capstone.

We were not trying to evaluate any of these teaching methods in isolation, and we have no way to disentangle their various contributions to our success or failure.

Our index of success was the extent to which the students were able to use the tools to do useful and reproducible work on a scientific question of their choosing. In § 2, we describe what we taught and how. In § 3, our main interest is an analysis of the work in the student projects. If we succeeded, then the student projects should be reproducible, and we should find good evidence that the students engaged with the tools, data, and scientific question.

## 2. Material and methods

We taught *Reproducible and Collaborative Statistical Data Science*,^3^ during the fall semester of 2015. The course was offered through the department of Statistics at UC Berkeley, and was open to upper-level undergraduates (as STAT 159) as well as graduate students (as STAT 259).

The course entry requirements were Berkeley courses STAT 133 (Concepts in Computing with Data), STAT 134 (Concepts of Probability), and STAT 135 (Concepts of Statistics). Together these courses provide basic undergraduate-level familiarity with probability, statistics, and statistical computing using the R language. Many students were from statistics and/or computer science; other majors included cognitive science, psychology, and architecture. During the 15-week long semester, we had three hours of class time per week in two 90-minute sessions, plus two hours of lab time. Students were expected to work at least eight hours per week outside class. Project reports were due in week 17, two weeks after the last class.

### 2.1. Course overview

This was the entry for our course in the Berkeley course catalog:

A project-based introduction to statistical data analysis. Through case studies, computer laboratories, and a term project, students learn practical techniques and tools for producing statistically sound and appropriate, reproducible, and verifiable computational answers to scientific questions. Course emphasizes version control, testing, process automation, code review, and collaborative programming. Software tools include Bash, Git, Python, and 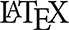.

#### 2.1.1. Tools and process

We had three guiding principles in our choice of tools and process: (a) to teach efficient reproducible practice with standard expert tools, (b) to teach fundamental mathematical and statistical principles using explanation with simple code, and (c) students should be able to build their own analysis from basic building blocks.

Applying these principles, we taught command line tools, version control, and document machinery using text files. Rather than focusing on a specific neuroimaging analysis package, we taught scientific coding with Python and its associated scientific libraries (Millman and Aivazis, 2011; Pérez et al., 2011), and then showed the students how to run standard statistical procedures on imaging data using these tools (Millman and Brett, 2007).

##### 2.1.1.1. Command line

The Unix environment is the computational equivalent of the scientists' workbench (Preeyanon et al., 2014). The Unix command line, and the Bash shell in particular, provides mature, well-documented tools for building and executing sequences of commands that are readable, repeatable, and can be stored in text files as scripts for later inspection and execution. Quoting Wilson et al. (2014)—the Bash shell makes it easier to “make the computer repeat tasks” and “save recent commands in a file for re-use.”

The graphical user interface of operating systems such as Windows and macOS can obscure the tree structure of the file system, making it harder to think about the organization of data as a hierarchy of directories and files. The command line tools make the file system hierarchy explicit, and so make it easier to reason about data organization.

##### 2.1.1.2. Version control

Version control is a fundamental tool for organizing and storing code and other products of data analysis. Distributed version control allows many people to work in parallel on the same set of files. The tools associated with distributed version control make it easier for collaborators to review each other's work and suggest changes.

Git is the distributed version control system that has become standard in open source scientific computing. It is widely used in industry, with automated installers for all major operating systems. Web platforms such as GitHub,^4^ Bitbucket,^5^ and GitLab^6^ provide web interfaces to version control that simplify standard methods of collaboration such as code review, automated testing, and issue tracking (see below).

##### 2.1.1.3. Scientific python

Python is a general purpose programming language, popular for teaching and prevalent in industry and academia. In science, it has particular strength in astronomy, computational biology, and data science. Its impact in scientific computing rests on a stack of popular scientific libraries including NumPy (computing with arrays), SciPy (scientific algorithms including optimization and image processing), Matplotlib (2D plotting), Scikit-Learn (machine learning), and Pandas (data science). The Nibabel library^7^ can load files in standard brain image formats as arrays for manipulation and display by the other packages.

##### 2.1.1.4. Peer review

Regular peer review is one of the most important ways of learning to be an effective author of correct code and valid data analysis. Git and its various web platforms provide a powerful tool for peer review.

*Pull requests* are a version control interface feature pioneered by GitHub. A pull request presents proposed changes to the shared repository in a convenient web interface. Collaborators can comment on the changes, ask questions, and suggest improvements. The discussion on this interface forms a record of decisions, and a justification for each accepted set of changes.

GitHub, like other hosting platforms for version control, provides an interface for creating *issues*. These can be reports of errors that need to be tracked, larger scale suggestions for changes, or items in a to-do list.

##### 2.1.1.5. Functions with tests

Functions are reusable blocks of code used to perform a specific task. They usually manipulate some input argument(s) and return some output result(s). There are several advantages to organizing code into functions rather than writing stream-of-consciousness scripts. Functions encapsulate the details of a discrete element of work. A top-level script may call a well-named function to express a particular step, allowing the user to “hide” the mechanism of this step in the function code. A top-level script that uses functions in this way gives a better overview of the structure of the analysis. Functions make it easier to reuse code at various points in the analysis, instead of rewriting or copying and editing it. Finally, writing functions allows the programmer to test small parts of the program in isolation from the rest.

Testing is an essential discipline to give some assurance to the authors and other users that the code works as expected. Tests should also document how the code is expected to be used. Inexperienced coders typically greatly underestimate how often they make errors; expecting, finding, and fixing errors is one of the foundations of learning from continued practice. There are tools that can measure what proportion of the lines of code are covered by the tests—this is *test coverage*.

##### 2.1.1.6. Continuous integration

Tests are useless if they are not run, or if the authors do not see the results. Modern code quality control includes *continuous integration testing*, a practice that guarantees that the tests are run frequently and automatically, and the results are reported back to the coding team. Fortunately, this practice has become so ubiquitous in open source, that there are large free services that implement continuous integration, such as Travis CI,^8^ Circle CI,^9^ and Appveyor.^10^ These services can integrate with hosting sites like GitHub in order to run tests after each change to the code.

##### 2.1.1.7. Markup languages

A markup language allows you to write content in plain text, while also maintaining a set of special syntax to annotate the content with structural information. In this way, the plain text or *source* files are human-readable and work well with standard version control systems. To produce the final document, the source files must undergo a *build* or *render* step where the markup syntax (and any associated style files) are passed to a tool that knows how to interpret and render the content.


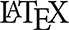
 and Markdown represent two extremes of markup languages, each with their own usefulness. 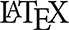 is notation-heavy, but powerful. In contrast, Markdown is notation-light, but limited.

Pandoc is a command line document processor that can convert between multiple markup formats. It can also generate rendered output from source files with text markup. For example, it can generate PDF files from text files written with Markdown markup.

##### 2.1.1.8. Reproducible workflows

When working with the Unix command line, we frequently generate files by performing a sequence of commands. The venerable Make system was originally written to automate the process of compiling and linking programs, but is now widely used to automate all types of command line workflows.

Makefiles are machine-readable text files consisting of rules specifying the sequence of commands necessary for generating certain files and for tracking dependencies between files. Consider the following Makefile rule:


progress.pdf: progress.md
 pandoc -t beamer -s progress.md -o progress.pdf


This rule defines the procedure to generate the PDF slide show file progress.pdf. The first line specifies the *target* of the rule, and any *prerequisites*. Here the target is progress.pdf; building this target requires the source Markdown text file progress.md. The indented line gives the *recipe*, which is the mechanism by which the target should be generated from the prerequisites. In this case, the recipe is to execute the pandoc utility on the Markdown file, with various options applied.^11^ If you edit progress.md, you can regenerate progress.pdf from the command line using make progress.pdf.

In the example above, the recipe was a single command, but Makefile recipes often involve several commands. The target of a rule is often a filename (e.g., progress.pdf), but can also be an arbitrary name to describe the sequence of commands in the recipe. The dependencies of a rule can be filenames (e.g., progress.md), but may also be other rules in the Makefile. These features allow Makefiles to chain together complex sequences of commands necessary for generating multiple target files, which may depend on other files or steps; such chaining is important for building and maintaining reproducible workflows.

#### 2.1.2. Lectures, labs, notes, homeworks, readings, and quizzes

The weekly lectures and labs prepared students with the skills and background needed for the group project. We demonstrated methods and tools during lecture and lab, and expected students to do additional research and reading as they worked on the group project. For example, we only gave one or two lectures on each of Unix, Git, Python, testing, and NumPy. Each weekly lab focused on the software stack from previous lectures. For example, after the Git lecture, we used Git and GitHub to fetch and submit all exercises; after introducing code testing in lecture, we continually reinforced testing in the labs. Course work outside labs also made heavy use of the toolstack, including each new tool as it was introduced in lectures.

For the first three lectures, we introduced students to the Unix environment and version control using Git. The fourth lecture was a high-level introduction to neuroimaging and functional magnetic resonance imaging (or FMRI). We then introduced students to scientific computing with Python. We referred students to our course notes on Bash,^12^ Git,^13^ Make,^14^ Python,^15^ scientific Python packages,^16^ and 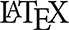.^17^

By the ninth lecture (week 5), we started to focus on the analysis of FMRI data with Python. Data analysis lectures continued to develop the students' skills with Unix and the scientific Python software stack through practical problems, which arose as we taught FMRI analysis methods.

We assigned two homeworks. Students had two weeks to work on each homework. Homeworks were assigned and submitted using Git and private GitHub repositories during the first half of the semester. Typically, assignments were given as a collection of tests that tested the desired behavior of named functions, and function templates with missing implementations. The functions were documented according to the NumPy documentation standard.^18^ Students added implementations to the functions according to the documentation and ran the tests to check that their code returned the correct results for the provided tests. Assignments were graded using extended tests, which were not provided to the students before they submitted their work. The homework reinforced the material we taught during the beginning of the course and focused on scientific programming in Python.

Over the course of the semester, seven readings were assigned on a roughly bi-weekly basis. The readings consisted of articles that emphasized the core concepts of the class, either with respect to scientific computing, or neuroimaging. Students were required to compose a two-paragraph write-up that both summarized the main points of the article, and commented on it.

The labs and quizzes emphasized hands-on experience with the computing tools and process associated with reproducible and collaborative computing (e.g., version control, 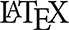, etc.). Multiple choice quizzes were held at the beginning of lab sessions, and emphasized the computing aspects of the course material as opposed to the statistical and neuroimaging components covered in the lectures. The remainder of the lab was devoted to providing hands-on experience via collaborative work on breakout exercises. These exercises were formulated as small projects to be worked on in groups of three to four students.

We also used the labs to help the students practice critical code review. For example, we had the students form small groups and review one another's solutions to the second homework. This involved all the students in each group creating pull requests to a shared GitHub repository with their solutions. Then they had to use the GitHub code review model, which we had been practicing in class, to review three functions in each solution. We asked them to summarize their findings with respect to clarity, brevity, and performance. We used their summaries to inform a subsequent discussion about code review.

### 2.2. Course project

The course centered around a semester-long group project that accounted for 55% of the overall course grade. Students worked on their projects in teams for three months.

Early in the semester (week 5) groups of three to five students formed teams. To make sure every aspect of the project work was computationally reproducible and collaborative, we immediately created a publicly visible project repository for each team. The groups defined the scope of their projects iteratively; the teams submitted their proposals in week 6, a third of the way through the semester. We gave each team feedback to clarify project motivation and goals.

**Figure 1:**
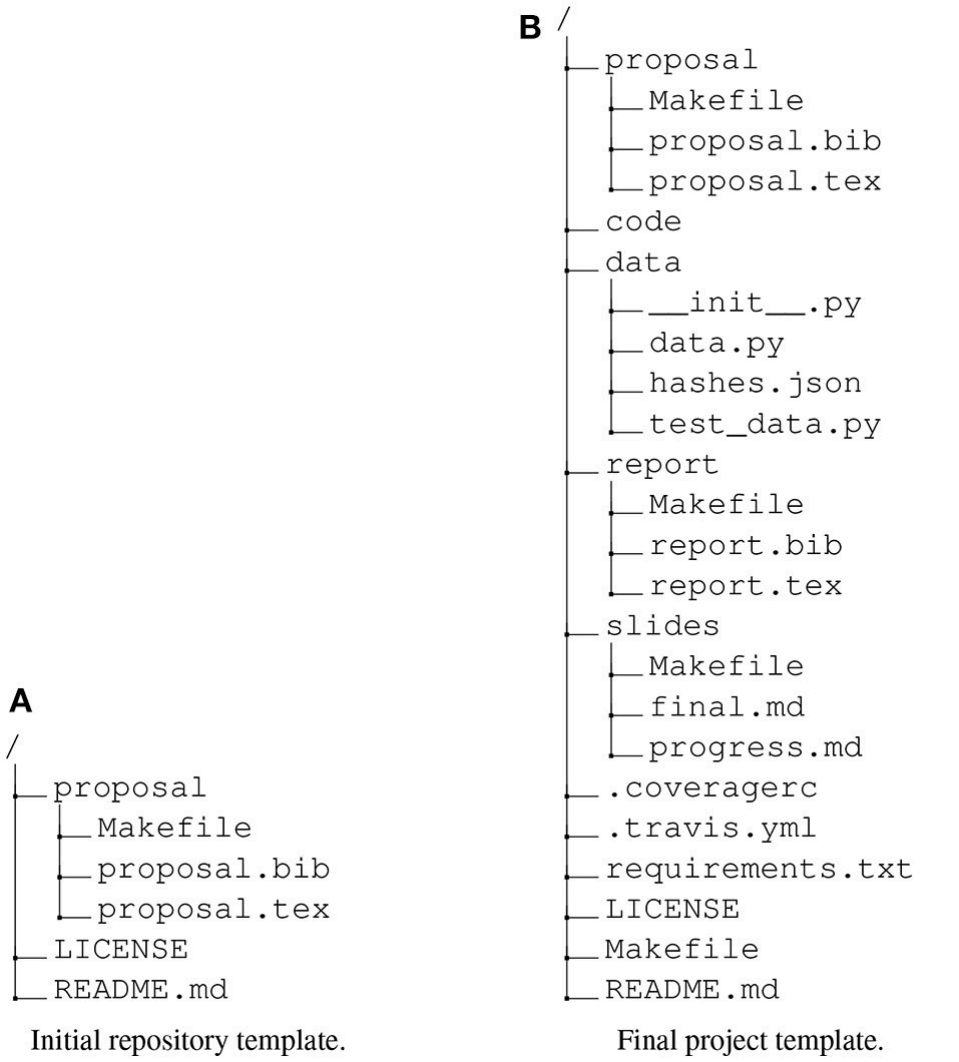
The initial and final repository directory template for student projects. We gave the students a project copied from the initial template in week 5, from which they would write and build and their initial proposal. As the course progressed, we pushed two updates to their repository. The first gave them a template for building their final report, their top-level README.md file and file specifying an open license for their work. The second gave them: an initial set of functions we had shown them in class with associated tests (in the code directory, not shown); machinery to trigger automatic execution of tests on Travis-CI servers; automated code coverage reporting; and a template for writing slides for their first progress report. **(A)** Initial repository template. **(B)** Final project template.

For their project proposals, we required the teams to use a 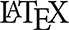 template, which we provided, and to add the source file(s) to their project repository (see Figure 1). Each proposal involved (a) identifying a published FMRI paper and the accompanying data, (b) explaining the basic idea of the paper in a paragraph, (c) confirming that they could download the data and that what they downloaded passed basic sanity checks (e.g., correct number of subjects), and (d) explaining what approach they intended to take for exploring the data and paper. All teams chose a paper and accompanying dataset from OpenFMRI,^19^ a publicly-available depository of MRI and EEG datasets (Poldrack et al., 2013; Poldrack and Gorgolewski, 2015).

We reviewed the project proposals and met with each team in week 7 to help them refine their ideas. The feedback process continued throughout the semester, with students regularly submitting drafts according to milestones defined within the project timeline. As much as possible, we used this process to replicate the way we work with collaborators in the lab.

Project feedback included peer-review, where each research team was responsible for cloning another team's project, running the tests and analyses, and evaluating the resulting technical document. The peer-review process proved particularly valuable, as the students benefited greatly from the exposure to the coding techniques and organizational principles of their peers.

Students used GitHub's pull request mechanism to review all project work. Code was written as a collection of functions (with tests) and short scripts that called these functions to perform the project data analysis. We taught students to test their code thoroughly. We set up each project with a configuration file for the Travis CI^8^ continuous integration service, and enabled integration of this service with their GitHub repository. The configuration file specified that their tests would be run and reported on the Travis CI servers each time they changed their code on the GitHub hosting site. We added automated code coverage testing; each change to the code on GitHub resulted in a measure of the proportion of all code lines covered by tests. We expected students to keep measured test coverage above 90%. While each team decided when to merge pull requests, we recommended that all pull requests should have at least three participants, test coverage should not decrease, and tests should pass before being merged.

Students also used the GitHub issues interface to record project discussions and actions taken by the group members. We told students to create a pull request or issue with code and text and use the GitHub interface to ask one or more of us to respond, if they had any questions about their projects. Periodically, we would review their work and open issues.

We covered this workflow in depth during lectures and in lab, and made clear that the project grade would be based on (a) the final report, (b) the analysis code, (c) whether we could run their analysis code to reproduce all their results and figures, and (d) how effectively the team collaborated using the techniques taught in the course (e.g., pull requests, code review, testing). We told the students that we would use the pull request and issue discussions as evidence for their contributions to the project, and as data for their final grades.

Rather than have students commit the raw FMRI data to their project repository, we had them commit code to download and validate the raw data used in their project (week 8). Since this was the first code they added to their repositories, we gave them example code to download a file and validate it by checking the file against a stored checksum of the file content. Once they added code to handle downloading and validating the data, we had them commit code for their exploratory data analysis (week 9). After this, the only milestones we provided were for progress presentations and report drafts.

**Figure 2:**
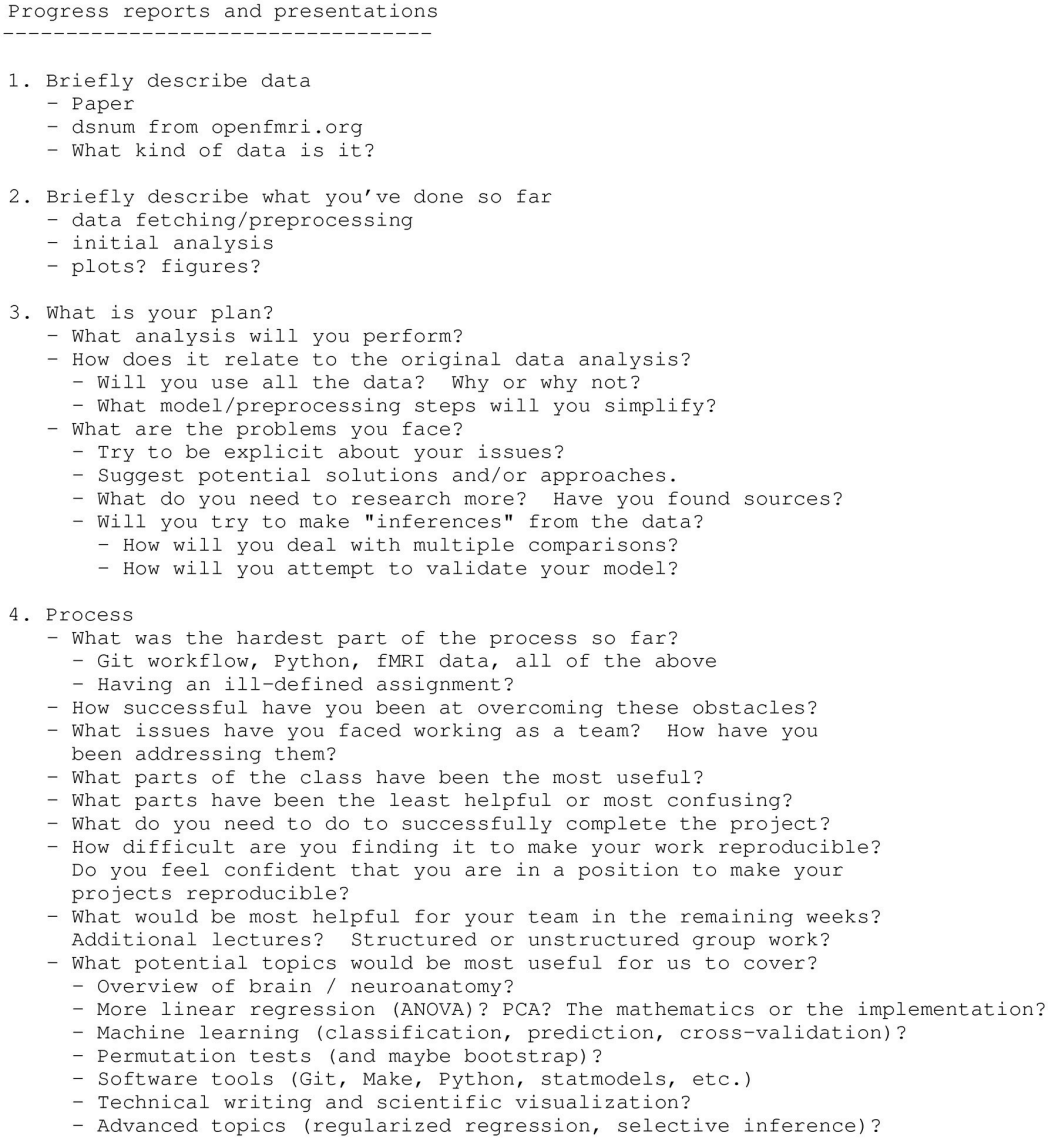
Class lecture material giving instructions for the first progress report. This is the text we showed them during class, and discussed, to prepare them for their first progress report. As for almost all our lecture material, we posted this text to the class website.

The students gave their first progress report and presentation in week 12; it consisted of a description of the data they were using, what they had done so far, their plan for completing the project, and a reflection on the overall process (see Figure 2). We required the groups to write their slides in Markdown text format for building into PDF with Pandoc. They committed the Markdown source to the project repository. The students gave their second slide presentation in week 15, two weeks before the projects were due, discussing their current progress and their plan for finishing their project. At the end of the project, in week 17, they had to commit the final PDF of their reports and provide instructions for generating the report PDF from the source 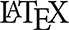 files.

Early in the project, we told the students that we would grade their project work on whether it was reproducible. In order to reproduce their work, we told the students we would blindly follow the instructions in a text file named README.md in the root directory of the project repository. When we graded their projects, the README.md file had to explain how to install, configure, and run all aspects of their project. Each README.md had to specify how to rebuild all the components of the project and in what order to do so. For example, it might include a section specifying the make commands to execute, such as:


make data         #  download the data
make validate   #  ensure the data is not corrupted
make eda          #  generate figures from exploratory analysis
make analysis   #  generate figures and results
make report      #  build final report


### 2.3. Neuroimaging data analysis

In terms of neuroimaging, our aims were for students to (a) understand the basic concepts in neuroimaging, and how they relate to the wider world of statistics, engineering, and computer science; (b) be comfortable working with neuroimaging data and code, so they could write their own basic algorithms, and understand other people's code; (c) continue to use the computational techniques we had taught them to improve efficiency and help their understanding.

For this course we concentrated on the statistical analysis of FMRI data using a linear model. We designed each lecture to teach the next analysis step the students would need for their project work.

All the following teaching used simple Python code to show them how the mathematics works in practice, and to give them sample code that they could edit and run for themselves.^20^ We specifically avoided using off-the-shelf imaging analysis software packages, and encouraged the students to build their own analyses from the building blocks we had given them. In lectures, we interleaved teaching with short group exercises where students took the code from class and extended it to solve a new problem.

We covered the following topics:

- The idea of images as visual displays of values in arrays.
- The standard neuroimaging NIfTI image format as a simple image container, combining metadata (such as shape) and image pixel/voxel data stored as a sequence of numbers. We used the Nibabel Python package^7^ to load NIfTI data as three-dimensional arrays.
- Four dimensional (4D) images as sequential time-series of 3D image volumes. Slicing arrays to get individual volumes from the 4D time-series. Extracting single-voxel time-courses from 4D images.
- Building regressors for the statistical model. We introduced the idea of the neuronal time-course model as the hypothesized change in neuronal activity caused by the task. For a block design, this model becomes a box-car, with all rest periods having identical low activity, and all activity periods having identical high activity. The FMRI acquisition measures something like blood flow, rather than neuronal activity, but blood flow changes slowly in response to changes in neuronal activity. We introduced the linear-time-invariant assumption in predicting the hemodynamic activity from the hypothesized neuronal activity, and then the hemodynamic response function as the invariant response. Finally we demonstrated the mechanism of convolution as a numerical method to express the effect of the hemodynamic response function acting in a linear-time-invariant way, and the caveats to these assumptions. We now had hemodynamic regressors to fit to our voxel time-course data.
- Correlation as a simple test of linear association between the hemodynamic regressor and voxel signal. Calculating correlation with a single regressor for every brain voxel and the idea of a statistical parametric map.
- The linear model as a matrix formulation of multiple regression. We started with simple (single-regressor) regression as another measure of linear association. We expressed simple regression as a linear model and showed how the model can be expressed as the addition of vectors and vector / scalar multiplication. This leads to the matrix formulation of simple regression, and thence to multiple regression. We introduce dummy indicator variables to express group membership and show how these relate to group means. We showed with code how this mathematics can express statistical methods that they already know, such as regression, *t*-tests, and ANOVA.
- High-resolution sampling of regressors. The simple cases that we had covered above assumed events or blocks that started at the same time as the scanner started to collect a new brain volume. This is not the case in general. To deal with events that do not start at volume onsets, we need to generate a hemodynamic time-course at higher time resolution than the spacing of the scan onsets, and then sample this regressor (using linear interpolation) at the scan onset times.
- Parametric modulation of event regressors. Some of the OpenFMRI datasets that the students had chosen used regressors to model parametric modulation of events. There is one regressor to model the hemodynamic effect of a particular event type on average, and another to capture the variation of the hemodynamic event activity as a linear function of some characteristic of that event, such as duration or intensity.
- Spatial smoothing with a Gaussian kernel; smoothing as a form of convolution; the scipy.ndimage subpackage in SciPy as an implementation of Gaussian and other smoothing.
- The idea of voxel and millimeter coordinates in brain images, and the image affine as a mapping between them. The students needed this in order to relate the coordinates reported in their papers to voxels in their own analyses.

**Figure 3:**
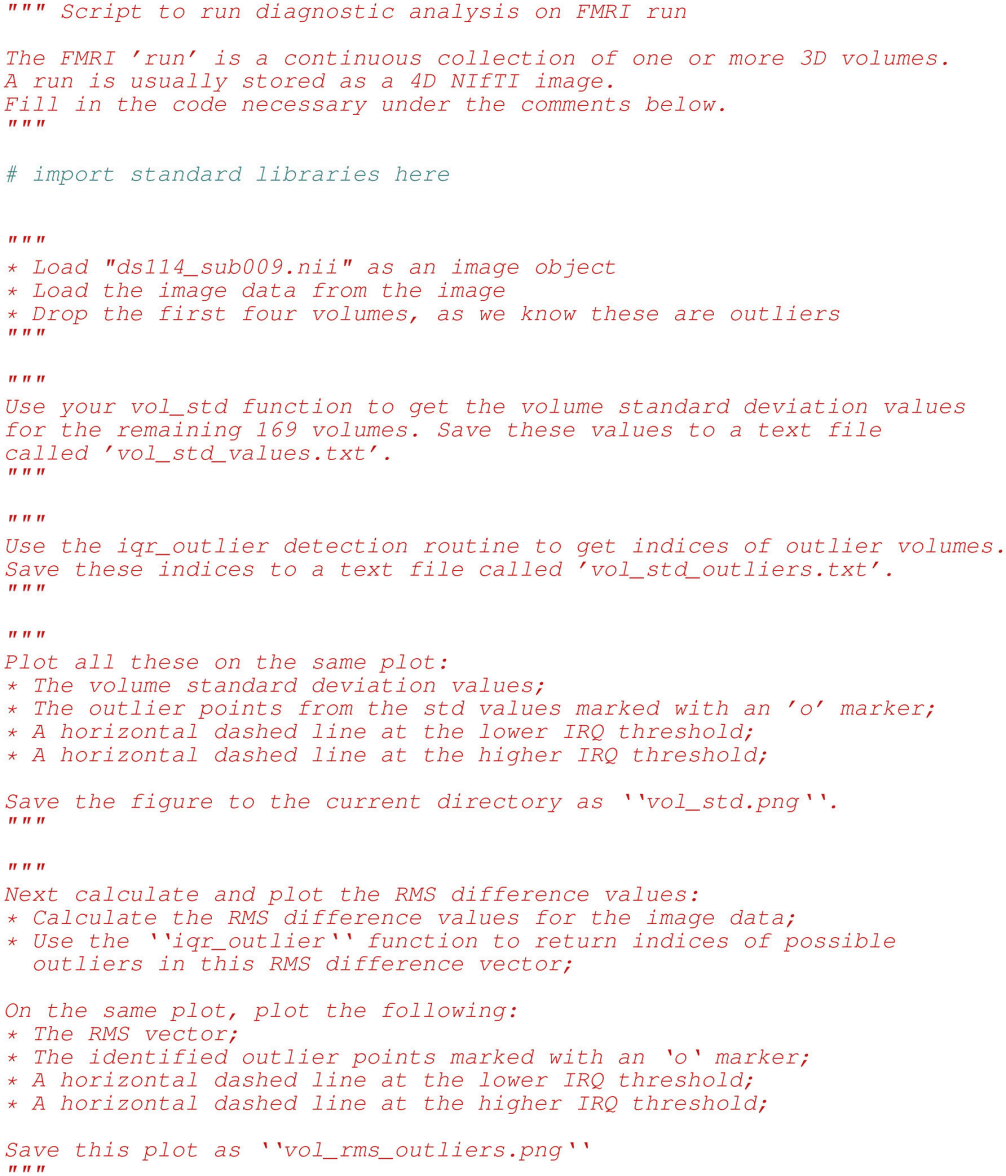
Above we list the first few lines of diagnosis_script.py. This file was part of the second homework, which focused on detecting outlier 3D volumes in a 4D FMRI image. Tasks included: **(a)** implementing functions on image arrays using NumPy, **(b)** exploring FMRI data for outliers, **(c)** running least-squares fits on different models, and **(d)** making and saving plots with Matplotlib.

We reinforced the material from class lectures and exercises in the homeworks. Figure 3 shows an excerpt from the second homework, in which we asked to students to fill out functions implementing various algorithms on 4D FMRI data and use these functions to build up a simple diagnostic test for outlier volumes in the time series. Finally, they applied a linear (multiple regression) model to the data to show that removing outlier scans reduced residual variance from the model fit.

We encouraged students to use the basic Python building blocks for their project analyses, but we did not insist. Our rule was that, if they used other software, they had to persuade us that they had a sound understanding of the algorithms that the other software was using.

### 2.4. Project grading

We based the evaluation of the final projects on criteria that emphasized the underlying principles of reproducibility, collaboration, and technical quality. See Table 1 for the final project grading rubric, which we gave to the students on week 11—the week before the first progress presentations. From the perspective of reproducibility, we evaluated projects on whether the presented results could be generated from their repositories according to the documentation they provided in their README.md text file. When we could not reproduce the analysis, we raised one or more GitHub issues to negotiate with the project team. We graded the code tests with respect to code coverage. Grading for the collaborative aspects of the project used the information from the project history provided by the Git version history and GitHub web artifacts, including code contributions, as well as reviewing GitHub pull requests and discussion on GitHub issues. Finally, we assessed the technical quality of the project in terms of the clarity of the final report and with respect to how well the proposed goals of the study were met by the final results of the analysis.

**Table 1:** Project grading rubric.

	**✓–**	**✓**	**✓+**
Questions	Questions overly simplistic, unrelated, or unmotivated	Questions appropriate, coherent, and motivated	Questions well motivated, interesting, insightful, and novel
Analysis	Choice of analysis overly simplistic or incomplete	Analysis appropriate	Analysis appropriate, complete, advanced, and informative
Results	Conclusions missing, incorrect, or not based on analysis	Conclusions relevant, but partially correct or partially complete	Relevant conclusions tied to analysis and context
	Inappropriate choice of plots; poorly labeled plots; plots missing	Plots convey information but lack context for interpretation	Plots convey information correctly with adequate and appropriate reference information
Collaboration	Few members contributed substantial effort or each members worked on only part of project	All members contributed substantial effort and everyone contributed to all aspects of project	All members contributed substantial effort to each project aspect
Tests	Tests incomplete, incorrect, or missing	Tests cover most of the project code	Extensive and comprehensive testing
Code review	Pull requests not adequately used, reviewed, or improved	Pull requests adequately used, reviewed, and improved	Code review substantial and extensive
Documentation	Poorly documented	Adequately documented	Well documented
Readability	Code readability inconsistent or poor	Code readability consistent and good quality	Code readability excellent
Organization	Poorly organized and structured repository	Reasonably organized and clear structure	Elegant and transparent code organization
Presentation	Verbal presentation illogical, incorrect, or incoherent	Verbal presentation partially correct but incomplete or unconvincing	Verbal presentation correct, complete, and convincing
	Visual presentation cluttered, disjoint, or illegible	Visual presentation is readable and clear	Visual presentation appealing, informative, and crisp
	Verbal and visual presentation unrelated	Verbal and visual presentation related	Verbal and visual presentation clearly related
Writing	Explanation illogical, incorrect, or incoherent	Explanation correct, complete, and convincing	Explanation correct, complete, convincing, and elegant
Reproduciblity	Code didn't run	Makefile recipes fetch data, validates fetched data, generates all results and figures in report	Makefiles generate EDA work and supplementary analysis

*An “A” was roughly two or more check pluses and no check minuses*.

## 3. Results

There were a total of eleven research teams composed of three to five students, each responsible for completing a final project of their own design using datasets available through the OpenFMRI organization. We named groups arbitrarily with Greek letters (e.g., alpha, kappa, zeta). The project repositories are public at https://github.com/berkeley-stat159.

Although we allowed students to select any OpenFMRI dataset, in fact all groups selected one of the following:

- ds000005:^21^ *Mixed-gambles task* (Tom et al., 2007) (groups delta, epsilon, eta, theta);
- ds000009:^22^ *The generality of self-control* (Cohen and Poldrack, 2014) (group alpha);
- ds000105:^23^ *Visual object recognition* (Haxby et al., 2001) (groups kappa, zeta);
- ds000113:^24^ *A high-resolution 7-Tesla FMRI dataset from complex natural stimulation with an audio movie* (Hanke et al., 2014) (groups beta, lambda);
- ds000115:^25^ *Working memory in healthy and schizophrenic individuals* (Repovš and Barch, 2012) (groups gamma, iota).

During grading, we succeeded in fully replicating all group analyses. The minor problems that arose were largely platform differences between macOS used by the students and the Linux system we were using for grading. Four projects reproduced without issue, six required us to raise one GitHub issue, and one required two issues.

Table 2 shows code metrics for the project repositories, and statistics for the projects' use of the GitHub interface.

**Table 2:** GitHub and other code metrics for student projects.

**Project**	**Commits**	**Issues**	**PRs**	**Comments**	**Words/comment**	**LoC**	**% Covered**
Alpha	787	23	190	379	24.7	3,293	3.7
Beta	534	7	147	105	20.1	1,753	2.0
Delta	571	31	121	117	35.1	996	21.3
Epsilon	593	26	310	79	40.9	1,809	19.5
Eta	259	11	89	44	21.7	588	12.4
Gamma	329	4	79	35	22.3	1,040	37.6
Iota	414	26	113	144	24.3	928	8.4
Kappa	337	30	99	86	16.6	1,157	3.5
Lambda	365	22	67	82	17.6	732	91.5
Theta	547	25	133	450	22.1	1,186	23.1
Zeta	344	3	49	21	20.8	8,287	6.2

Commits: *number of Git commits in repository*. Issues: *number of GitHub issues*. PRs: *number of GitHub Pull Requests*. Comments: *number of comments, either on issues or PRs, not including the initial description of the issue / PR*. Words / comment: *mean number of words per comment*. LoC: *total lines of code measured with http://cloc.sourceforge.net, not including test directories*. % covered: *Estimate of percentage of lines of code in* LoC *column that were covered by tests, from statistics at https://coveralls.io. Counts for commits, issues, PRs and comments do not include those created by instructors, or those added after the end of semester. See https://github.com/jarrodmillman/rcsds-paper/tree/master/project_metrics for details of these calculations*.

The median final lines of non-test code per project was 1,157. We were not successful in persuading the students to test a large proportion of their code; we estimated that tests covered 12.4% of code lines with a range of 2% to 91.5%. The code coverage machinery we had put in place did not measure code lines outside expected code directories, and the students had put a large proportion of their code outside these directories, leaving the automated code coverage score above 90% in all cases, even though a large proportion of the code was not in fact covered by tests.

The GitHub statistics suggest that the teams did engage with Git and GitHub workflow. All projects used the Pull Request (PR) feature to a reasonable degree, with a median of 113 PRs per project. There was considerable variation in the extent to which the teams used comments to review PRs and issues, with a median of 86 comments per project. All projects except Zeta had a low ratio of final lines of code to number of commits, ranging from 1.7 to 4.2. Zeta appears to have used a different strategy, with the most lines of code per commit (24.1), and the smallest number of comments.

No team used SPM, FSL, or AFNI. Two projects imported the nilearn package,^26^ which is a Python neuroimaging package designed for machine learning, but both projects used Nilearn only for spatial smoothing and basic volume visualization. One project used a single function from the Dipy package^27^ for calculating a within-brain mask for the functional images. Another used a class from the NiTime package^28^ for temporal filtering. All projects used the basic NumPy, SciPy, and Matplotlib packages; eight of eleven used the Scikit-Learn package^29^ for machine learning; six used the Statsmodels package^30^ for statistical modeling. The uses of Statsmodels were: AutoRegressive Integrated Moving Average (ARIMA) modeling of voxel time-series and autocorrelation plots (two projects); logistic regression (two projects); ordinary least squares modeling (one project); White test for Heteroscedasticity (one project); mixed effects modeling (one project). Of these, we had only shown the implementation of ordinary least squares analysis in class.

There was a wide range in the novelty and scope of the projects. Most projects consisted of a serious attempt to replicate one or several statistical findings in the original paper, with the addition of some further extension or exploration. These extensions were typically the application of other statistical techniques. For example, project epsilon was one of the four groups working on the dataset ds000005—the *Mixed-gambles task*. They first explored the imaging data using the outlier detection machinery that all students had developed in the second homework. Next they explored various logistic regression models of the behavioral data. For the imaging data, they used the SciPy ndimage subpackage to smooth the data, as we had briefly shown in class, and then followed some hints in the lectures to explore different confound models such as linear and quadratic drift, and Principal Components as regressors. They used code from class to implement the general linear model at each voxel, and calculate *t*- and *p*-values using contrast vectors. Finally they thresholded their images using Bonferroni correction.

Other projects did more substantial technical or intellectual extensions of the original analysis. Project alpha explored an analysis from ds000009—*The generality of self-control*. Although we had not covered this in class, they discovered from data exploration and reflection that the times of slice acquisition would affect their statistical modeling, and developed code to shift their model back and forth in time corresponding to the time of slice acquisition. They tried various confound models on the data, including linear drift, Fourier bases and different numbers of PCA regressors, and explored these models with selection criteria such as the Bayes and Akaike Information Criteria, and adjusted *R*^2^. They ran diagnostics to detect voxels violating assumptions of normality. For whole brain analysis, they implemented the FDR multiple comparison correction and tried other methods for identifying activated brain regions, including hierarchical clustering. Finally, they experimented with ARIMA models of the voxel time course.

Project lambda did an heroic effort to replicate the analysis of ds000113—a high-resolution FMRI dataset of subjects listening to a description of the film Forrest Gump. This dataset had many technical challenges; the images are unusually large, at over 1 million voxels per volume, and of unusually long duration, at around 450 volumes per run, and having 8 runs per subject. The analysis requires the correlation of many voxel time-courses in each subject with voxel time-courses in all other subjects in the analysis. The group did a variety of explorations, including finding some artifacts in the original data, and an error in the published paper, and then went on to replicate the original correlation analysis, on a smaller number of subjects. They ran the analysis on Amazon Web Services machines in order to deal with the demands of processing time and memory. As they note in their README.md “We strongly encourage running on a machine with 120 GBs of accessible RAM to emulate development environment.” They extended the analysis by running a random forest model to detect volumes corresponding to outdoor and indoor scenes in the film. With all this, they achieved around 92% code test coverage.

In the next paragraph, we report scores from the student course evaluations. We consider them to be a measure of student satisfaction. Student ratings have little or no relationship to the success of the course in conveying the material (Boring et al., 2016; Uttl et al., 2017), but they do appear to measure a variety of unfortunate and irrelevant factors such as the gender and race of the teacher (Boring et al., 2016), the subject being taught (Uttl and Smibert, 2017), and the grade the student expects to get at the end of the course (Krautmann and Sander, 1999; Worthington, 2002).

Seventeen out of forty undergraduates and six out of ten graduate students completed anonymous course evaluations. The primary question of interest to us was “Considering both the limitations and possibilities of the subject matter and the course, how would you rate the overall effectiveness of this course?.” Ratings were on a 1 through 7 scale with 4 corresponding to “moderately effective” and 7 corresponding to “extremely effective.” The average undergraduate and graduate scores were 4.71 and 6.0 respectively, against a department average across all courses of 5.23. Undergraduate, graduate, and department average ratings for “I enjoyed this class” on a 1 through 7 scale (with 4 corresponding to “somewhat” and 7 to “very”) were 4.69, 6.00, and 4.96 respectively.

70.5% of the undergraduate respondents and all the graduate respondents claimed to have worked 10 or more hours a week on average.

## 4. Discussion

Most neuroimaging researchers agree that computational reproducibility is desirable, but rare. How should we adapt our teaching to make reproducibility more common?

The usual practical answer to this question, is that we should train neuroimaging and other computational researchers as we do now, but add short courses or bootcamps that teach a subset of the tools we taught here but without the substantial practice. That is, reproducibility is an addition on top of current training.

We believe this approach is doomed to failure, because the students are building on an insecure foundation. Once you have learned to work in an informal and undisciplined way, it is difficult to switch to a process that initially demands much more effort and thought. Rigor and clarity are hard to retrofit. To quote the American chemist Frank Westheimer: “A couple of months in the laboratory can frequently save a couple of hours in the library.”

For these reasons, our course took the opposite approach. We put a substantial, collaborative, and open-ended project at the center of the course. We then started with the tools and process for working with numerical data, and for building their own analyses. We used this framework as a foundation on which to build their understanding of the underlying ideas. As we taught these tools, we integrated them into their exercises and homework, and made it clear how they related to their project work.

Our claim is that this made our teaching and our students much more efficient. The secure foundation made it easier for them to work with us and with each other. As they started their project work, early in the course, they could already see the value of these tools for clarity and collaboration. Our students graduated from the course with significant experience of using the same tools that experts use for sound and reproducible research.

### 4.1. Did we really teach neuroimaging analysis?

By design, our course covered tools and process as well as neuroimaging. We used neuroimaging as the example scientific application of these tools. Can we claim to have taught a meaningful amount of neuroimaging in this class?

Class content specific to neuroimaging was a guest lecture in class 4 (of 25), and teaching on the brain images, correlation, and the general linear model from classes 9 through 15. We covered only the standard statistical techniques needed for a basic analysis of a single run of FMRI data. This is a much narrower range than a standard neuroimaging course, but we covered these topics in much greater depth than is typical for a neuroimaging course. This was the basis for final projects that were substantial and well-grounded.

Typical imaging courses do not attempt to teach the fundamental ideas of linear models, but assume this understanding and move onto imaging specifics. This assumption is almost entirely false for neuroscience and psychology students, and largely false for our own students, even though most had training from an undergraduate statistics major. As a result of this incorrect assumption, it is rare for students of neuroimaging to be confident in their understanding of the statistics they are using. We taught the linear model from the first principles of vector algebra, using the tools they had just learned, to build a simple analysis from basic components. As a result, when the students got to their projects, they had the tools they needed to build their own neuroimaging analysis code, demonstrating and advancing their own understanding.

We note the difficulty of the task that we gave the students, and the extent of their success. We made clear that their project was an open-ended exploration of an FMRI dataset and paper. Few students had any experience or knowledge of FMRI before the course. The only guidance we gave was that they should prefer well-curated datasets from the OpenFMRI depository. In order to design and implement their project, they had to understand at least one published FMRI paper in some depth, with limited assistance from their instructors. We gave no example projects for them to review, or templates for them to follow. The submitted projects were all serious efforts to reproduce and / or extend the published results, and all included analysis code that they had written themselves.

We did not teach the full range of neuroimaging analysis. For example, we did not cover pre-processing of the data, random effects analysis, or inference with multiple comparisons. Our claim is that what we did teach was a sound and sufficient foundation from which they could write code to implement their own analyses. This is a level of competence that few neuroimagers achieve after considerable experience.

### 4.2. Did we really teach computational reproducibility?

We stressed the importance of reproducibility throughout the course, and made it an explicit feature for grading of the final project, so we were not surprised to find that it was possible to reproduce all of the final projects with little extra clarification or fixes from the group members.

By design, we gave the students a project that was close to a real scientific data analysis. They had to work with a messy and real dataset to explore the data, define their problem and solve it with an analysis that they implemented themselves, to various degrees. We required them to work closely in teams, often remotely, using standard tools for collaboration.

We found this combination of a substantial analysis problem, expert tools, and the requirement for reproducibility, was effective at giving the students a concrete sense of the difficulties in making an analysis reproducible, and how these can be overcome. We speculate, from our own work, that the experience of building a reproducible analysis makes it easier to commit to the tools and practice needed for reproducible work in the future.

### 4.3. Did we teach the right tools?

We do not believe that the individual tools we chose were controversial. We taught the tools that we use ourselves, and that we would teach to students working with us.

We could have used R instead of Python, but Python is a better language for teaching, and has better libraries for neuroimaging and machine learning.

A more interesting question is whether we went too far in forcing the students to use expert tools. For example, we required them to write their report in 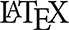, and their presentation slides and analysis description in plain text with Markdown markup. We asked them to do all project interaction using GitHub tools such as the issue tracker and pull requests. We set and marked code exercises with Git version control and text files, rather than interactive alternatives, such as Jupyter Notebooks.

Of course—at first—some students complained that they would rather use Facebook for code discussions and PowerPoint for their presentation slides. Should we have allowed this? We believe not. Our own experience of using these tools is that their power only becomes apparent when you learn to use them for all the tasks of analysis, including generating reports and presentations. Mixing “easy” but heavy tools like PowerPoint and Facebook with “simple” and light tools like text editors and Markdown causes us to lose concentration as we switch modes from heavy to light and back again. It is easy to be put off by the difficulty of getting used to the light tools, and therefore fail to notice the clarity of thought and transparency of process that they quickly bring. Successfully switching from heavy to light is a process that requires patience and support; it is best done in the context of a class where there are examples of use from coursework and support from experienced instructors who use these tools in their daily work.

### 4.4. Do students need to be trained in programming?

One common justification for not training students in good coding practice is the assertion that scientists do not need to be programmers. However, scientists do have to use, read, and write code, so a more defensible statement would be that scientists do not need to be *good* programmers. It is surely true that successful scientists can be bad programmers, but bad programmers are inefficient and prone to error; they are less likely to detect errors, and improve very slowly over time. We should invest teaching time to help our students work efficiently and continue learning for the rest of their careers.

### 4.5. Do students need a substantial, collaborative, and open-ended project?

We put great emphasis on the final project in this course, and this was clear to most of the students. In response to the evaluation survey question “*What advice would you give to another student who is considering taking this course?”* one undergraduate wrote:

“[U]nlike most group projects (which last for maybe a few weeks tops or could conceivably be pulled off by one very dedicated person), this one will dominate the entire semester. . . . Try to stay organized for the project and create lots of little goals and checkpoints. You should always be working on something for the project, whether that's coding, reviewing, writing, etc. Ask lots of questions and ask them early!”

The size of the project meant that the students had to learn to collaborate with each other efficiently, often remotely, as students had different class schedules. Many of the tools that we taught, such as distributed version control, only become essential when working in collaboration. Conversely, if you are not collaborating with others, it can be difficult to see why it is worth investing the time to understand powerful tools like the Git version control software, or the GitHub interface.

Working in collaboration, and working reproducibly, changes the way that we think. If we have to explain our work to others in the group, or to another user of the work, then we develop the expectation that whatever we write will always be something we will demonstrate and explain to others. It becomes part of the work to communicate our ideas and explain what we did.

It was important that the project was open-ended. If the student has to solve a small problem with a single correct answer, they can often check whether they have the right answer, and do not need to worry about the quality of the process that generated it. In an open-ended project, it is likely that the group will need to explore different analyses as they progress. The answers are not known, and the group has to proceed with care, to avoid making false conclusions. This is typical of real scientific analysis, and puts a higher burden on rigor and testing, than a typical small classroom problem.

We should emphasize how hard it was to get the students to engage with the project early, and work steadily. In the first class, we gave the students a document describing the project, including the various project deadlines. We continued to emphasize the project and project deadlines in announcements. Nevertheless, it was only half way through the course that the students began to realize how open-ended their task was, and how much work they would have to do. For a few weeks, the class was anxious, and our job as instructors was to project faith in the teams' ability to define a tractable and interesting question. We mention this to say that, a large open-ended project has many advantages, but to make it work, it does take courage from the students and the instructors.

### 4.6. What background do students need?

The requirements for our course were previous classes on probability, statistics, and the use of the R programming language.

We would be happy to relax the requirement for probability. We did not refer to the ideas of probability in any depth during the course. Authors JBP and MB have taught similar material to neuroscience and psychology students who lack training in probability; the pace of teaching and level of understanding were similar for the statistics students in this course and the other students we have taught.

Psychology and neuroscience students do have some statistical training. Our impression was that this background was necessary for us to be able to as start as quickly as we did in the analysis of linear regression.

We would also keep the requirement for some programming experience. We assumed familiarity with programming ideas such as for loops, conditionals, and functions. In our psychology / neuroscience courses that used similar material, we required some experience of programming in a language such as Python, R, or MATLAB. It is possible that a brief introduction would be enough to fulfill this requirement, such as a bootcamp.

### 4.7. Could this material be covered by a bootcamp or hack week?

There are several existing programs designed to address the problems of informal and inefficient computational practice. The best known may be Software Carpentry (Wilson, 2014). A Software Carpentry course consists of a two-day bootcamp covering many of the same tools we used, including the Unix command line, distributed version control, and programming with a high-level language such as Python or R. Hack weeks can be a related approach to the same problems. Hack weeks vary in format from a series of tutorials to a week of collaborative work on a project that has already been agreed (Huppenkothen et al., 2017). The tutorials and modeled working practice usually include the same set of tools we used.

These approaches have some similarity with the early classes in our course, but there are differences. Bootcamps and hack weeks attract volunteers with interest in or commitment to efficient and reproducible practice. These are typically graduate students or post-docs that already have some technical experience. They are motivated enough to come to campus on a weekend, stay longer at a conference, or travel some distance. Even so, we suspect that it would be possible to show that short introductions in bootcamps will not be effective in changing practice in the medium term, without later reinforcement and support by peers.

Our students did choose our course from others they could have taken, but most of them had little background of good practice in computation. The majority were undergraduates. We suspect that many of the students finishing our course did have enough experience of using the tools they had used, to continue using them in their daily work, and teach others to do the same. We base this suspicion on the depth and quality of the project work.

Bootcamps and hack weeks can be useful, but their starting point is an attempt to augment an aging curriculum that does not recognize the need for training in accurate, effective, and reproducible computation. We should fix this by changing our curriculum; learning to work this way takes time, practice, and support, and we teach it best with substantial commitment from students and instructors.

### 4.8. Where would such a course fit in a curriculum?

Our course would not fully qualify a student for independent research in neuroimaging. As we discuss above, we did not cover important aspects of imaging, including spatial and temporal pre-processing of data, random effects, or control of false positives in the presence of multiple comparisons. Where should the elements of our course sit in relation to a larger program for training imagers and other computational scientists?

We think of this course as a foundation. Students graduating from this course will be more effective in learning and analysis. The tools that they have learned allow the instructor to build analyses from basic blocks, to show how algorithms work, and make them simple to extend. We suggest that a course like ours should be first in a sequence, where the following courses would continue to use these tools for exposition and for project work.

A full course on brain imaging might start with an introduction to Python and data analysis, possibly as a week-long bootcamp at the start of the semester. Something like our course would follow. There should be follow-up courses using the same tools for more advanced topics such as machine learning, spatial pre-processing, and analysis of other modalities. We suggest that each of these courses should have group project work as a large component, in which the students continue to practice techniques for efficient reproducible research.

### 4.9. What factors would influence the success of such a course?

We should note that there were factors in the relative success of this course that may not apply in other institutions.

Berkeley has as a strong tradition in statistical computing and reproducibility. Leo Breiman was a professor in the Berkeley statistics department, and an early advocate of what we would now call data science. He put a heavy emphasis on computational teaching to statisticians. Sandrine Dudoit is a professor in the statistics and biostatistics departments, and a founding core developer of the Bioconductor project devoted to reproducible genomics research in R. The then head of the statistics department, Philip Stark, is one of many Berkeley authors (including KJM) to contribute to the recent book “The Practice of Reproducible Research” (Kitzes et al., 2017). Authors KJM, MB, and JBP have worked with Mark D'Esposito, who runs the Berkeley Brain Imaging Center, and was an early advocate for data sharing in FMRI. Other imaging labs on campus take this issue seriously and transmit this to their students. If there had not been such local interest in the problem of reproducibility, students may have been less convinced of the importance of working in a reproducible way, especially given the short-term convenience of a less disciplined working process.

Students of statistics and other disciplines at Berkeley are well aware of the importance of Python in scientific computing, and in industry. Tech firms recruit aggressively on campus, and Python is a valuable skill in industry. The cross-discipline introductory course in Fundamentals of Data Science uses Python. The new Berkeley Institute of Data Science has a strong emphasis on Python for scientific computing.

In the same way, a booming tech sector nearby made it more obvious to students that they would need to learn tools like Git and GitHub. For example, public activity on GitHub is one feature that companies use to identify candidates for well-paid positions as software engineers.

We required students to use 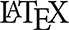 to write their final reports. This was an easy sell to statistics students, but students outside mathematical fields might be less agreeable; if we were teaching psychology and neuroscience students, we might choose another plain text markup language, such as Markdown, with the text files run through the Pandoc document processor to write publication quality output.

### 4.10. Instruction or discovery?

We have asserted from our experience, that very few neuroimaging researchers do research that is computationally reproducible. We believe this is because researchers are left to work out—discover—their analysis process with little guidance or feedback from scientists and programmers with greater experience and training. The current state of neuroimaging practice is reminiscent of the failures of “pure-discovery” and “minimal-guidance” learning (Mayer, 2004; Kirschner et al., 2006). Students that are early in their learning need expert guidance to be successful in building valid models of mathematics, logical puzzles, and programming. Although we did not know this literature when we designed our course, we did have the strong intuition that we would save the students a great deal of wasted time and energy by teaching the process that we had come to by a combination of long experience, reflection on failure, and learning from our peers in open source computing.

“Project-based learning” is another branch of educational theory and practice that uses projects to encourage students to discover knowledge through exploration of a “driving question.” Project-based learning is difficult to define (Condliffe, 2017), but an influential review by Thomas (2000) draws a distinction between “application” projects and project based learning. In project-based learning, “students encounter and learn the central concepts of the discipline via the project.” In contrast, application projects are those in which “project work follows traditional instruction in such a way that the project serves to provide illustrations, examples, additional practice, or practical applications for material taught initially by other means.” The projects in our course were application projects; we taught the techniques and principles, with the project as their practical application. Knoll (2012) traces the history of application projects from the early eighteenth century, when schools of architecture used projects as evidence that their students could apply the principles they had been taught in lectures and tutorials.

## 5. Conclusion

Our course differs from other imaging courses that we know of, in several respects. We started with correct computational practice, before teaching neuroimaging. We emphasized computation as a way of explaining the underlying ideas and taught the fundamentals of the linear model, rather than assuming students had this background, or would get this background later. We made these ideas concrete with a substantial open-ended project designed to be as close as possible to the experience of graduate research.

We were largely successful in teaching the students the tools they can continue to use productively for collaborative and reproducible research.

We are sure that most of our readers would agree that, in an ideal world, we should teach students to work in this way, but, given all the other classes our students must take, can we justify the time and energy that this course needs? We believe so, but we know that not all our readers will be convinced. This is true of students as well as instructors. We put such emphasis on the final project, precisely because we know that it is difficult to see how important these tools are, if you have not used them, or used them only in toy projects. We cannot easily teach these tools to our fellow teachers, so instead we offer this sketch of an experiment, to make the discussion more concrete. Imagine we took 200 students, and randomized them into two groups of 100 each. The first group takes something like the course we describe here as an introduction, and then one or more further courses on imaging. The second does not take such a course, but instead has more traditional teaching on imaging that covers a wider range of techniques, using off the shelf imaging software. Part of their later teaching would include some techniques for reproducible research. Two years after such an experiment, we predict that students from the first group will have a greater understanding of what they are doing, will be more effective in analysis, and more likely to experiment with new ideas. We think it much more likely that the first group will be doing reproducible research.

## Author contributions

KM was the lead instructor and was responsible for the syllabus and project timeline. KM and MB were responsible for lectures and created homework assignments. KM and RB were responsible for labs, readings, quizzes, and grading. All authors held weekly office hours to assist students with their projects. KM and MB wrote the first draft of the manuscript. All authors wrote sections of the manuscript, contributed to manuscript revision as well as read and approved the submitted version.

### Conflict of interest statement

The authors declare that the research was conducted in the absence of any commercial or financial relationships that could be construed as a potential conflict of interest.

## Footnotes

^1^Following Buckheit and Donoho (1995), we define an analysis as *computationally reproducible* when someone other than the original author of an analysis can produce on their own computer the reported results using the author's data, code, and instructions.

^2^http://archives.caltech.edu/pictures/1.10-29.jpg

^3^http://www.jarrodmillman.com/stat159-fall2015/

^4^https://github.com

^5^https://bitbucket.org

^6^https://about.gitlab.com

^7^http://nipy.org/nibabel

^8^https://travis-ci.org

^9^https://circleci.com

^10^https://www.appveyor.com

^11^http://pandoc.org/MANUAL.html#producing-slide-shows-with-pandoc

^12^http://www.jarrodmillman.com/rcsds/standard/bash.html

^13^http://www.jarrodmillman.com/rcsds/standard/git-intro.html

^14^https://www.youtube.com/watch?v=-Cp3jBBHQBE

^15^http://www.jarrodmillman.com/rcsds/standard/python.html

^16^http://www.scipy-lectures.org/

^17^https://www.youtube.com/watch?v=8khoelwmMwo

^18^https://github.com/numpy/numpy/blob/master/doc/HOWTO_DOCUMENT.rst.txt

^19^https://www.openfmri.org/

^20^For an example lesson showing our use of Python code to illustrate the mathematical and statistical ideas, see http://www.jarrodmillman.com/rcsds/lectures/glm_intro.html.

^21^https://openfmri.org/dataset/ds000005

^22^https://openfmri.org/dataset/ds000009

^23^https://openfmri.org/dataset/ds000105

^24^https://openfmri.org/dataset/ds000113

^25^https://openfmri.org/dataset/ds000115

^26^https://nilearn.github.io

^27^Dipy is a package for analysis of diffusion imaging data: http://nipy.org/dipy

^28^NiTime is a library for time-series analysis of data from neuroscience experiments: http://nipy.org/nitime

^29^http://scikit-learn.org

^30^http://www.statsmodels.org
